# Exposure to phthalates among premenstrual girls from rural and urban Gharbiah, Egypt: A pilot exposure assessment study

**DOI:** 10.1186/1476-069X-10-40

**Published:** 2011-05-16

**Authors:** Justin A Colacino, Amr S Soliman, Antonia M Calafat, Muna S Nahar, Adrienne Van Zomeren-Dohm, Ahmed Hablas, Ibrahim A Seifeldin, Laura S Rozek, Dana C Dolinoy

**Affiliations:** 1 Department of Environmental Health Sciences, School of Public Health, University of Michigan, Ann Arbor, Michigan, USA; 2 Center for Global Health, University of Michigan, Ann Arbor, Michigan, USA; 3 Department of Epidemiology, School of Public Health, University of Michigan, Ann Arbor, Michigan, USA; 4 National Center for Environmental Health, Centers for Disease Control and Prevention, Atlanta, Georgia, USA; 5 Tanta Cancer Center and the Gharbiah Cancer Society, Gharbiah, Egypt

## Abstract

**Background:**

Phthalates have been identified as endocrine active compounds associated with developmental and reproductive toxicity. The exposure to phthalates in premenstrual Egyptian females remains unknown. The objective of this study was to characterize phthalate exposure of a potentially vulnerable population of premenstrual girls from urban and rural Egypt.

**Materials and methods:**

We collected one spot urine sample from 60 10-13 year old females, 30 from rural Egypt, and 30 from urban Egypt from July to October 2009. Samples were analyzed for 11 phthalate metabolites. Additionally, we collected anthropometrics as well as questionnaire data concerning food storage behaviors, cooking practices, and cosmetic use. Phthalate metabolite concentrations were compared between urban and rural Egyptians as well as to age and gender matched Americans.

**Results:**

Monoethyl phthalate (MEP), was detected at the highest concentration in urine of Egyptian girls (median: 43.2 ng/mL in rural, 98.8 ng/mL in urban). Concentrations of urinary metabolites of di-(2-ethylhexyl) phthalate and dibutyl phthalate were comparable between Egyptians and age matched US girls. Storage of food in plastic containers was a statistically significant predictor of urinary mono-isobutyl phthalate (MiBP) concentrations when comparing covariate adjusted means.

**Conclusions:**

Urinary concentrations of phthalate metabolites were similar in Egyptian and US populations, suggesting that phthalate exposure also occurs in developing nations. Dietary intake is likely an important route of exposure to phthalates in both urban and rural populations.

## Background

Phthalates are a family of chemicals that have a wide range of applications in consumer goods, including children's toys, building materials, food packaging, cosmetics, cleaning materials, pharmaceuticals, and medical devices [1]. The molecular weight of the phthalate typically will determine in which application the compound is utilized. Higher molecular weight phthalates, such as di-(2-ethylhexyl) phthalate (DEHP), are most often used as plasticizers in polyvinyl chloride (PVC), while lower molecular weight phthalates, such as diethyl phthalate (DEP) and dimethyl phthalate (DMP), are used in cosmetics, insecticides, and pharmaceutical applications [2]. Phthalates, when used as plasticizers, are not chemically bound to PVC, hence they can leach from consumer products into air or food [1]. Diet is an important route of phthalate exposure; however, exposure can occur from a number of sources, including medical interventions, medications, inhalation of volatile phthalates, and from dermal application of phthalate containing personal care products [2-5].

Phthalates are chemicals of concern because of both their widespread use as well as documented toxic effects measured *in vitro, in vivo*, and in epidemiological studies. Studies conducted by the Centers for Disease Control and Prevention (CDC) have detected metabolites of DEHP and diethyl phthalate (DEP) in the majority of the US population [6-8]. Typically, children have higher urinary concentrations of several phthalate metabolites compared to adults [6,7,9,10]. Exposure of rats to high doses of phthalates orally (0.5 - 2 g/kg/day) has been associated with a range of health outcomes including prolonged estrous cycles and mid-pregnancy abortion [11,12]. Epidemiological studies of exposure to phthalates also suggest potential developmental and reproductive associations. A case control study conducted in Northern Mexico found significantly higher levels of monoethyl phthalate (MEP), a metabolite of diethyl phthalate (DEP), in breast cancer cases compared to controls [13], while evidence for phthalates' association with premature puberty and breast development is conflicting. Maternal urinary concentrations of phthalate metabolites, particularly monobutyl phthalate (MBP) and mono-(2-ethylhexyl) phthalate (MEHP), was associated with preterm birth in a Mexico City cohort [14]. Exposure to high molecular weight phthalates has been weakly associated with early breast and pubic hair development, while exposure to low molecular weight phthalates was weakly associated with later breast and pubic hair development [15].

Our group, among others, has described heterogeneity in disease rates between the urban and rural populations of Egypt [16,17]. Soliman and colleagues note higher ER-positive breast cancer incidence in urban versus rural Egyptian women, and overall higher rates of uterine, cervical, and breast cancer in the urban setting in the Nile delta of Egypt [16,17]. These differences in incidence rates may be due, in part, to xenobiotic chemical exposure at critical exposure time points such as pubertal development. Developing countries with gradients of exposure offer an interesting setting in which to study the association between toxicants and disease. However, it is important to understand the types and levels of toxicants in order to appropriately address potential health effects to the population.

In this pilot study, we measured the urinary concentrations of 11 phthalate metabolites in 60 premenstrual girls from urban and rural Egypt and compared them to United States population levels as measured in the 2003-2004 and 2005-2006 National Health and Nutrition Examination Survey (NHANES). Additionally, we evaluated the association between concentrations of urinary phthalate metabolites, anthropometric measures, and questionnaire data regarding potential routes of exposure including food and beverage storage, cooking practices, and practice of personal hygiene.

## Methods

### Subject Recruitment

We recruited healthy urban (N = 30) and rural (N = 30) premenstrual girls from primary schools from the Gharbiah province of Egypt between July and October 2009. Gharbiah province is an administrative region located 90 kilometers north of Cairo in the center of the Nile Delta Region, and has eight districts each with a capital city. Tanta serves as the capital city of the Tanta district as well as of the entire Gharbiah province. We assigned each participant a residence code based on their residential address that followed the Central Agency for Public Mobilization and Statistics (CAPMAS) national census coding of urban and rural areas. Urban girls were chosen by systematic random sample of girls from the census records of Tanta city as the urban location. Rural girls were chosen by a systematic random sample from two villages from two districts in the province. Study subjects were approached to participate in the study at local schools and no refusals were encountered. All study subjects were provided bus transportation to the Tanta Cancer Center. We obtained written informed consent from the mother of each study participant. Approval from the Institutional Review Boards of University of Michigan and the Gharbiah Cancer Society were obtained before starting the study. The involvement of the CDC laboratory was limited and determined not to constitute engagement in research on human subjects.

### Sample Collection

At the time of recruitment, the study subjects provided one urine sample, completed an interviewer administered questionnaire, and were measured for height, weight, waist, and hip circumference. We collected nine mL of urine in sterile polypropylene containers provided by the CDC between 12:00 and 4:00 PM to minimize temporal variability. We split urine samples into two aliquots, which were frozen and shipped, on dry ice, to the National Center for Environmental Health (NCEH) of the CDC and the University of Michigan School of Public Health. The questionnaire, entitled "Comparison of Xenoestrogen Levels Among Prepubertal Females in Urban and Rural Gharbiah, Egypt," contained discrete questions regarding residential history, personal care product usage, and family history including history of cancer, detergent usage, and food preparation and storage behaviors.

### Chemical Analyses

Scientists at the Division of Laboratory Sciences of NCEH, CDC determined the urinary concentrations of 11 phthalate metabolites: mono-carboxyisononyl phthalate (MCNP), mono-carboxyisooctyl phthalate (MCOP), mono-n-butyl phthalate (MBP), monoethyl phthalate (MEP), mono-(2-ethylhexyl) phthalate (MEHP), monobenzyl phthalate (MBzP), mono-(3-carboxypropyl) phthalate (MCPP), mono-(2-ethyl-5-hydroxyhexyl) phthalate (MEHHP), mono-(2-ethyl-5-oxohexyl) phthalate (MEOHP), mono-isobutyl phthalate (MiBP), and mono-(2-ethyl-5-carboxypentyl) phthalate (MECPP), by a modification of methods described previously [18]. Briefly, the analytical approach involved enzymatic deconjugation of the metabolites from their glucuronidated form, solid-phase extraction, separation with high performance liquid chromatography, and detection by isotope-dilution tandem mass spectrometry as described previously [18]. Isotopically-labeled internal standards and conjugated internal standards were used to increase precision of measurements. The limits of detection (LOD) ranged between 0.2 ng/mL to 1.2 ng/mL, depending on the phthalate metabolite. Along with the unknown samples, each analytical run included calibration standards, reagent blanks, and quality control (QC) materials, prepared from urine pools spiked with high and low concentration of the target phthalate metabolites, to monitor for accuracy and precision. The urinary concentrations of the phthalate metabolites in the QC materials--averaged to obtain one measurement of high-concentration QC and one of low-concentration QC for each batch--were evaluated by using standard statistical probability rules [19]. CDC personnel had no access to information about the participants.

### Data Analyses

Phthalate measurements below the LOD were estimated as the LOD divided by the square root of two. Specific gravity adjustment was conducted as described previously [14]. Adjusted urinary phthalate metabolite levels were calculated by the formula P_c _= P[(1.018 - 1)/(SG-1)] where P_c _is the specific gravity adjusted phthalate metabolite concentration (ng/mL), 1.018 is the sample population median specific gravity value, and SG is the measured specific gravity for each urine sample. Three samples had specific gravity values less than 1.005. These samples were excluded from the analyses because of the urinary sample being too dilute, adapting previously established criteria for male workers to this young female population [20]. However, including these samples in the analysis of the unadjusted urinary concentration of phthalate metabolites did not significantly alter the results.

Demographic and anthropometric variables analyzed included age, body mass index, waist circumference, hip circumference, urban/rural status, use of plastic containers in food storage, use of plastic food utensils in cooking or eating, and canned food consumption. Univariate statistics were used to describe mean, median, standard deviations and ranges of the demographics, anthropometrics, and chemical measurements and were calculated in R 2.9.2 (R Development Core Team, 2010).

We extracted an age and gender matched subset of the National Health and Nutrition Examination Survey (NHANES) for the years 2003-2006 [21] for comparison of urinary phthalate metabolite concentrations with the Egyptian study subjects. Additionally, we restricted our NHANES analysis to individuals who participated in the afternoon session, to most closely match the time of day when we collected urine from the Egyptian study subjects. Concentrations of urinary phthalate metabolites were available for 97 comparison subjects, except for MCNP and MCOP, where concentrations were available for 44 subjects because MCNP and MCOP were not measured in NHANES 2003-2004. We calculated univariate summary statistics for the NHANES data using survey procedures in SAS software (version 9.2, SAS Institute, Cary, NC) using the relevant strata, clusters, and weights.

Comparisons of concentrations of urinary phthalate metabolites between urban and rural Egyptians were conducted via Wilcoxon rank sum test, as log transformation of the phthalate metabolite data did not yield a normal distribution. Additionally, comparisons between concentrations of urinary phthalate metabolites between individuals who reported storing/not storing food in plastic containers as well as individuals who reported using/not using plastic utensils were assessed with the Wilcoxon rank sum test. Associations between anthropometric measures (weight, hip circumference, and waist circumference) and urinary concentrations of phthalate metabolites were estimated using Spearman rank correlations. Associations between urban/rural status and food storage and consumption behaviors were estimated via Pearson's Chi-squared test. Covariate adjusted least squares means and standard errors of phthalate metabolite concentrations in urine were calculated using PROC GLM and the LSMEANS statement in SAS 9.2. Means were adjusted for age, BMI, and urinary specific gravity. Adjusted means between urban and rural individuals, as well as individuals who reported storing food in plastic containers or not, were compared by t-test, with a p < 0.05 considered statistically significant.

## Results

The study subjects ranged in age from 10-13 years with an average age of 11.5 years. There was no difference in age or body composition between subjects recruited from rural or urban areas (Table 1). There was no significant association between anthropometrics and urinary phthalate metabolite concentrations by Spearman rank correlations. There was no statistically significant difference in unadjusted concentrations of individual urinary phthalate metabolites measured between urban and rural study subjects (Table 2). The individual phthalate metabolite measured at the highest concentration was MEP, a metabolite of diethyl phthalate (DEP), with a median concentration of 98.8 ng/mL in urban individuals and 40.4 ng/mL in rural individuals. Girls in both the urban and rural groups had MEP urinary concentrations above the 95^th ^percentile of the sample population, signifying that the highest exposed individuals in the population were distributed in both urban and rural areas. Urinary concentrations of MECPP, a DEHP metabolite, were the highest among the four DEHP metabolites measured, and were also similar between urban and rural study subjects. Urinary specific gravity levels were significantly higher in rural compared to urban Egyptians by the Wilcoxon rank-sum test (p = 0.043) and specific gravity adjusted urinary concentrations of three phthalate metabolites, MBzP, MCPP, and MiBP, were found to be significantly higher in urban individuals (Table 3). Individuals who reported storing food in plastic bags or containers were found to have significantly higher specific gravity adjusted concentrations of MiBP by the Wilcoxon rank sum test (p = 0.01). Individuals who reported consuming canned food had significantly higher levels of MiBP (p-value = 0.02) and mEHP (p-value = 0.04). There were no significant differences in urinary phthalate metabolite concentrations observed in individuals who reported using plastic utensils or plastic plates.

**Table 1:** Age, anthropometric measurements, and food storage and dietary habits of the study subjects.

	Urban Egyptians (n = 28)			Rural Egyptians (n = 29)		
	**Mean (S.D.)**	**Median**	**Range**	**Mean (S.D.)**	**Median**	**Range**

Age (years)	11.4 (0.9)	11.4	10.1-13.2	11.6 (0.9)	11.6	10.1-13.6
Body Mass Index (BMI)	20.2 (4.0)	18.6	13.8-30.3	19.3 (4.0)	19.1	13.7-31.2
Waist Circumference (cm)	61.6 (11.3)	60.1	35.5-92.0	59.9 (7.5)	59.3	48.5-80.0
Hip Circumference (cm)	42.9 (6.9)	42	31.0-62.0	40.4 (4.9)	40.8	33.0-56.3

	Yes (%)			Yes (%)		

Reported food storage in plastic	23 (82%)			20 (69%)		
Reported using plastic utensils*	17 (61%)			25 (86%)		
Reported consuming canned foods*	19 (68%)			7 (25%)**		

S.D. - Standard Deviation;*significantly different by urban/rural status (p < 0.01);** one non-respondent (n = 28)

**Table 2:** Unadjusted urinary concentrations (in ng/mL) of phthalate metabolites in Egyptian girls and U.S. population as measured in the National Health and Nutrition Examination Survey (NHANES) 2003-2006

	NHANES 2003-2006^a^			Urban Egyptians (n = 28)			Rural Egyptians (n = 29)		
**Metabolite**	**Mean (S.E.)**	**Median**	**Range**	**Mean (S.E.)**	**Median**	**Range**	**Mean (S.E.)**	**Median**	**Range**

Mono-carboxyisononyl phthalate (MCNP)	7.3 (1.1)	5.0	0.4-31.8	1.5 (0.2)	1	0.4-5.3	1.7 (0.4)	1.2	0.4-9.5
Mono-carboxy-isooctyl phthalate (MCOP)	12.7 (3.1)	7.0	0.5-72.2	5.1 (1.1)	3.2	0.5-25.5	3.5 (0.5)	2.5	0.5-11.5
Mono-n-butyl phthalate (MBP)	54.5 (9.54)	25.7	1.5-466.6	94.7 (18.8)	53.3	5-375	86.7 (28.0)	47.5	3.5-809
Mono-ethyl phthalate (MEP)	461.7 (114.7)	114.9	5.3-18990	399.2 (272.9)	98.8	9.7-7740	400.0 (188.1)	43.2	3.8-4290
Mono-(2-ethylhexyl) phthalate (MEHP)	5.6 (0.7)	2.5	0.6-46.6	7.2 (1.5)	4.7	0.9-26.8	5.6 (1.2)	3.5	0.85-24.8
Mono-benzyl phthalate (MBzP)	34.7 (2.8)	25.1	0.4-393.6	4.4 (1.2)	2.2	0.2-28.1	2.0 (0.6)	0.4	0.2-16.7
Mono-(3-carboxypropyl) phthalate (MCPP)	5.7 (0.6)	3.6	0.1-61.1	2.9 (0.6)	2	0.2-12.7	1.9 (0.5)	1	0.1-12.2
Mono-(2-ethyl-5-hydroxyhexyl) phthalate (MEHHP)	48.3 (4.73)	29.1	0.7-423.7	48.6 (11.2)	29.1	1.1-263	30.5 (4.7)	23	2.6-104
Mono-(2-ethyl-5-oxohexyl) phthalate (MEOHP)	34.3 (3.3)	19.9	0.5-318.5	33.0 (7.4)	18.8	0.5-173	22.3 (4.3)	16	1-107
Mono-isobutyl phthalate (MiBP)	13.7 (2.0)	4.8	0.2-411.1	45.0 (8.7)	25.4	0.7-184	37.2 (11.4)	17.6	1.1-258
Mono-(2-ethyl-5-carboxypentyl) phthalate (MECPP)	80.9 (8.7)	43.1	1.9-784.8	89.6 (18.8)	58	4.1-492	84.67 (14.8)	60.9	9.5-398

a- Adjusted for survey weights; n = 97 except for MCOP and MCNP where n = 44

S.E. - Standard Error of the Mean

**Table 3:** Specific gravity adjusted urinary concentrations (in ng/mL) of phthalate metabolites in Egyptian girls.

	Urban Egyptians (n = 28)			Rural Egyptians (n = 29)		
**Metabolite**	**Mean (S.E.)**	**Median**	**Range**	**Mean (S.E.)**	**Median**	**Range**

Mono-carboxyisononyl phthalate (MCNP)	2.6 (0.6)	1.5	0.3-11.2	2.3 (0.6)	1.1	0.2-13.7
Mono-carboxy-isooctyl phthalate (MCOP)	9.0 (2.3)	3.8	0.4-49.0	4.0 (0.9)	2.7	0.6-24.5
Mono-n-butyl phthalate (MBP)	171.3 (54.5)	69.7	9-1350	82.3 (26.5)	43.2	3.2-728.1
Mono-ethyl phthalate (MEP)	562.2 (331.2)	121.8	8.8-9288	498.4 (239.5)	63	4.6-6462
Mono-(2-ethylhexyl) phthalate (MEHP)	13.6 (4.2)	4.6	0.6-94.0	5.4 (1.3)	3.1	0.4-29.6
Mono-benzyl phthalate (MBzP)*	9.2 (4.0)	2.4	0.2-101.2	1.8 (0.4)	0.8	0.1-7.5
Mono-(3-carboxypropyl) phthalate (MCPP)*	5.3 (1.7)	2.2	0.2-45.7	2.1 (0.6)	1.1	0.1-14.6
Mono-(2-ethyl-5-hydroxyhexyl) phthalate (MEHHP)	79.9 (20.5)	31	2.6-347.0	31.2 (4.7)	23.8	3.1-99.4
Mono-(2-ethyl-5-oxohexyl) phthalate (MEOHP)	54.6 (14.4)	21.7	0.9-257.8	20.9 (2.9)	14.4	1.2-52.8
Mono-isobutyl phthalate (MiBP)**	68.6 (17.6)	33.2	2.5-475.2	34.5 (10.1)	13.7	1.2-232.2
Mono-(2-ethyl-5-carboxypentyl) phthalate (MECPP)	154.5 (37.5)	74.7	14.8-644.4	82.6 (11.4)	68.6	10.2-249.5

Specific Gravity	1.015 (0.009)	1.015	1.005-1.035	1.021 (0.010)	1.020	1.005-1.040

S.E. - Standard Error of the Mean

*p-value < 0.05 by Wilcoxon rank-sum test of difference between Urban vs. Rural concentrations

**p-value < 0.01 by Wilcoxon rank-sum test of difference between Urban vs. Rural concentrations

When comparing concentrations of phthalate metabolites in urine measured in the Egyptian study population to age, gender, and urine collection time matched US population levels from NHANES, some differences were noted. The median US concentrations of MBzP were considerably higher (25.1 ng/mL) than those seen in either urban (2.0 ng/mL) or rural (0.3 ng/mL) Egyptians, and the maximum concentrations detected in the Egyptians did not approach the US median concentrations. The urinary concentrations of the four DEHP metabolites measured in this study, MEHP, MEOHP, MEHHP, and MECPP, were similar between the US and rural and urban Egyptians. Additionally, urinary concentrations of MEP and MBP were similar in the two populations. Egyptians, however, had higher median concentrations of MiBP, with urban (25.0 ng/mL) and rural (17.5 ng/mL) exceeding the US concentrations (4.8 ng/mL) at least threefold.

Least squares adjusted means and standard errors of urinary phthalate metabolites were calculated, adjusting for specific gravity, age, and BMI (Table 4). Adjusted mean phthalate metabolite concentrations were compared between urban and rural Egyptians as well as between individuals who reported storing food in plastic containers against those who did not. There were no statistically significant differences in covariate adjusted mean concentrations between urban and rural Egyptians. Individuals who reported storing food in plastic containers had significantly higher urinary concentrations of MiBP when comparing covariate adjusted means (p = 0.04). Additionally, while only MiBP concentrations were found to be statistically significantly higher in individuals reporting food storage in plastic, adjusted mean concentrations were found to be higher for every phthalate metabolite measured.

**Table 4:** Least squares adjusted mean urinary phthalate metabolite concentrations (ng/mL), adjusted for age, BMI, and urinary specific gravity.

	Area of Residence				Reported Food Storage in Plastic			
	**Rural (n = 29)**		**Urban (n = 28)**		**No (n = 14)**		**Yes (n = 43)**	

**Metabolite**	**Mean**	**S.E**.	**Mean**	**S.E**.	**Mean**	**S.E**.	**Mean**	**S.E**.

Mono-carboxyisononyl phthalate (MCNP)	1.7	0.3	1.2	0.4	1.2	0.4	1.7	0.2
Mono-carboxy-isooctyl phthalate (MCOP)	3.1	0.9	4.4	1.0	2.7	1.3	4.8	0.7
Mono-n-butyl phthalate (MBP)	66.5	25.5	81.0	29.0	42.2	35.2	105.2	19.7
Mono-ethyl phthalate (MEP)	370.1	241.2	262.5	273.7	154.5	333.0	478.1	186.3
Mono-(2-ethylhexyl) phthalate (MEHP)	4.6	1.4	6.3	1.6	3.5	2.0	7.3	1.1
Mono-benzyl phthalate (MBzP)	1.9	1.0	3.6	1.2	1.9	1.4	3.6	0.8
Mono-(3-carboxypropyl) phthalate (MCPP)	1.7	0.6	2.3	0.6	1.2	0.8	2.8	0.4
Mono-(2-ethyl-5-hydroxyhexyl) phthalate (MEHHP)	24.8	8.9	45.5	10.1	26.4	12.3	43.9	6.9
Mono-(2-ethyl-5-oxohexyl) phthalate (MEOHP)	17.8	6.3	30.5	7.1	17.3	8.6	31.0	4.8
Mono-isobutyl phthalate (MiBP)	28.4	10.7	36.3	12.1	15.2*	14.7	49.5*	8.2
Mono-(2-ethyl-5-carboxypentyl) phthalate (MECPP)	71.0	17.3	79.3	19.7	53.8	24.0	96.5	13.4

S.E. - Standard Error

* - Significantly different by t-test (p = 0.04)

## Discussion

This study provides the first assessment of exposure to several phthalates among a group of Egyptian pre-adolescent girls in relation to their urban/rural status as well as lifestyle behaviors such as food storage in plastic. Although several studies suggest diet as the primary source of phthalate exposure [4,22-24], there are limited studies investigating and comparing phthalate exposure in various countries. Quantifying exposure to endocrine active compounds in developing countries is an important step to understanding how these compounds may impact the health of potentially vulnerable populations.

Concentrations of urinary phthalate metabolites in urban and rural Egyptian girls were fairly similar in comparison to those reported for age-adjusted Americans girls. The major differences appear with MBzP and MiBP. Urinary concentrations of MBzP in both urban and rural Egyptian girls were lower, while the MiBP urinary concentrations among the Egyptian groups were three fold higher, compared to their American counterparts. A study of phthalate metabolites measured in urine collected from pregnant women in Peru found significantly lower creatinine adjusted concentrations of MBzP, MCPP, MEP, and MiBP compared to pregnant US women [25]. A recent study of a group of pregnant women from Jerusalem, Israel, however, observed a different pattern of exposure when compared to US women, with MBzP urinary concentrations lower and MiBP levels higher in Israeli women [26]. The findings of the Israel study are similar to the present study, where median MBzP levels were lower but MiBP levels were higher in Egyptian girls compared to US girls. These differences may reflect social and behavioral variances in daily lifestyle between northern African/Middle Eastern and American cultures. Fewer benzyl butyl phthalate (BzBP) containing products such as vinyl flooring may contribute to comparatively low urinary concentrations of MBzP in Egyptian girls. Elevated MiBP concentrations in Egyptians compared to US individuals suggest differential routes of exposure to DiBP, a phthalate commonly used as a plasticizer as well as in inks and paints. Since individuals who reported consuming canned foods as well as storing food in plastic had significantly higher concentrations of MiBP in their urine, food storage and consumption could potentially explain this difference between the Egyptian and US populations. Urinary concentrations of other phthalate metabolites, however, in general do not significantly differ between individuals from Egypt and the US, implicating similar routes of exposure and contact with phthalate-containing products between American and Egyptian girls.

Similar to previous studies, these data indicate that storage of food in plastics is a relevant route of phthalate exposure in Egypt [27]. With globalization and widespread distribution of phthalate-containing products, contamination in developing countries will become a greater concern and it will be necessary to address manufacturing regulation at the international level.

Considering phthalate's use in a variety of products ranging from toys to pharmaceuticals, the extent of exposure to different phthalate esters will depend on the availability and application in the local community [2]. In a study concerning levels of urinary phthalate metabolites in samples from a US reference population collected from 1988-1994, rural females appeared to have higher urinary concentrations of MBzP, a metabolite of BzBP, compared to their male and urban counterparts [28]. In our study, urban individuals had a higher concentration of specific gravity adjusted MBzP than rural individuals, although a significant relationship was not observed for unadjusted concentrations. In India, urban men had significantly higher levels of DEP, DBP, and DEHP compared to rural men as well as decreased reproductive endpoints, potentially explained by a more widespread use of plastic products in urban areas [29]. In the current study, urban individuals also had significantly higher levels of specific gravity adjusted MiBP, a metabolite of di-isobutyl phthalate, but this relationship was not observed in unadjusted levels. The similar urinary concentrations of MECPP and other DEHP metabolites found in both urban and rural girls may suggest similar routes of exposure. Widespread urbanization and easy accessibility to phthalate-containing products may be closing the gap between rural and urban lifestyles and variable non-occupational exposure to phthalates as well as other environmental contaminants.

Food storage in plastics is becoming ubiquitous in many areas that once utilized ceramic and clay containers, since plastics are inexpensive and widely manufactured. When covariate adjusted means were compared for all measured metabolites, individuals who reported storing food in plastic containers had significantly higher urinary concentrations of MiBP. Therefore lifestyle behaviors such as diet and food storage among girls in Gharbiah, Egypt can directly explain sources of phthalate exposure more than geographical location. When a quantitative analysis was conducted on the amount of phthalates in foodstuffs, traditional hot Egyptian food such as Koushary and Foul Medams served in plastic bags contained detectable levels of the plasticizers DEHP and di-(2-ethylhexyl) adipate [27]. The amount of phthalates leaching into these warmly served delicacies stored in plastic dishes, cups, and bags depended on the temperature, plastic contact time, as well as fat content. Therefore the type of food stored in plastics may contribute to variable amounts of phthalate exposures in individuals.

The current study has several limitations. First, the relatively small sample size limits the conclusions about the distribution of urinary concentrations of phthalate metabolites between urban and rural Egyptians. The sample size limits the complexity of statistical analyses that could be performed. Additionally, we only measured phthalate metabolites in prepubescent females, who may have a different exposure profile than adult Egyptians. In this initial study, we collected questionnaire data that we thought would elucidate major pathways of phthalate exposure in this population, including food storage behaviors, cooking methods, cosmetic use, cleaning supply use, and residential history. Despite identifying that storage of food in plastic is significantly associated with urinary concentrations of phthalate metabolites, our lack of comprehensive dietary intake data limits our ability to pinpoint specific foods that may be contaminated with phthalates.

## Conclusions

In this study, we noted differences in urinary concentrations of phthalate metabolites by food storage. Additionally, we noted differences in the urinary concentrations of certain phthalate metabolites, MBzP, MCPP, and MiBP, between urban and rural individuals when these concentrations were adjusted for urinary dilution using specific gravity. However, these differences were not observed in unadjusted concentrations. We also note few differences in the urinary concentrations of specific phthalate metabolites between Egyptian and US girls, perhaps explained by routes/sources of exposure unique to a specific locale. This suggests that exposure to phthalates may be of equal concern in some developing countries as in the US. However, we conclude that phthalate exposure alone likely will not explain differences in health effects, including breast cancer, between these urban and rural women in Egypt. A comprehensive exposure assessment that includes pesticide exposure, heavy metals, diet, and air pollution, among other factors, will be necessary to explain environmental influences on health in this population.

## List of abbreviations

BzBP: Benzyl butyl phthalate; BMI: Body mass index; CAPMAS: Central Agency for Public Mobilization and Statistics; DBP: Di-*n*-butyl phthalate; DEHP: Di-(2-ethylhexyl) phthalate; DEP: Diethyl Phthalate; DiBP: Diisobutyl phthalate; MBP: Mono-n-butyl phthalate; MBzP: Monobenzyl phthalate; MCNP Mono-carboxyisononyl phthalate; MCOP: Mono-carboxyisooctyl phthalate; MCPP: Mono-(3-carboxypropyl) phthalate; MECPP: Mono-(2-ethyl-5-carboxypentyl) phthalate; MEHHP: Mono-(2-ethyl-5-hydroxyhexyl) phthalate; MEHP: Mono-(2-ethylhexyl) phthalate; MEOHP:Mono-(2-ethyl-5-oxohexyl) phthalate; MEP: Monoethyl phthalate; MiBP: Mono-isobutyl phthalate; MMP: Monomethyl phthalate; NCEH: National Center for Environmental Health; NCHS: National Center for Health Statistics; NHANES: National Health and Nutrition Examination Survey; PVC: Polyvinyl chloride.

## Competing interests

The authors declare that they have no competing interests.

## Authors' contributions

DCD, LSR, and ASS conceived and participated in the design of the study. IAS and AH coordinated and conducted field collection. DCD, MSN, and AVZ were responsible for sample management. AMC conducted the laboratory analysis. JAC and LSR performed statistical analyses. JAC drafted the manuscript, and all authors provided input for and approved the final version of the manuscript.
